# Knowledge gaps in patients with COPD and their proxies

**DOI:** 10.1186/s12890-017-0481-8

**Published:** 2017-10-30

**Authors:** Nienke Nakken, Daisy J. A. Janssen, Esther H. A. van den Bogaart, Jean W. M. Muris, Jan H. Vercoulen, Frank L. Custers, Gerben P. Bootsma, Michiel H. M. Gronenschild, Emiel F. M. Wouters, Martijn A. Spruit

**Affiliations:** 1 Department of Research & Education, CIRO, Horn, the Netherlands; 2 Centre of Expertise for Palliative Care, Maastricht University Medical Centre+ (MUMC+), Maastricht, the Netherlands; 3 Department of Family Medicine, CAPHRI School of Public Health and Primary Care, Maastricht University, Maastricht, The Netherlands; 4 Department of Medical Psychology and Department of Pulmonary Diseases, Radboud University Nijmegen Medical Centre, Nijmegen, the Netherlands; 5 Department of Respiratory Medicine, Zuyderland, Heerlen, the Netherlands; 6 Department of Respiratory Medicine, Maastricht University Medical Centre+ (MUMC+), Maastricht, the Netherlands; 7 Department of Respiratory Medicine, NUTRIM School of Nutrition and Translational Research in Metabolism, Maastricht University Medical Centre+ (MUMC+), Maastricht, The Netherlands

**Keywords:** COPD, Education, Family, Informal carer, Knowledge, Proxy

## Abstract

**Background:**

Although proxies of patients with chronic obstructive pulmonary disease (COPD) need health-related knowledge to support patients in managing their disease, their current level of knowledge remains unknown. We aimed to compare health-related knowledge (generic and COPD-related knowledge) between patients with COPD and their resident proxies.

**Methods:**

In this cross-sectional study, we included stable patients with moderate to very severe COPD and their resident proxies (*n* = 194 couples). Thirty-four statements about generic health and COPD-related topics were assessed in patients and proxies separately. Statements could be answered by ‘true’, ‘false’, or ‘do not know’. This study is approved by the Medical Research Ethics Committees United (MEC-U), the Netherlands (NL42721.060.12/M12–1280).

**Results:**

Patients answered on average 17% of the statements incorrect, and 19% with ‘do not know’. The same figure (19%) for the incorrect and unknown statements was shown by proxies. Patients who attended pulmonary rehabilitation previously answered more statements correct (about three) compared to patients who did not attend pulmonary rehabilitation. More correct answers were reported by: younger patients, patients with a higher level of education, patients who previously participated in pulmonary rehabilitation, patients with better cognitive functioning, and patients with a COPD diagnosis longer ago.

**Conclusions:**

Proxies of patients with COPD as well as patients themselves answer about two third of 34 knowledge statements about COPD correct. So, both patients and proxies seem to have an incomplete knowledge about COPD and general health. Therefore, education about general health and COPD should be offered to all subgroups of patients with COPD and their proxies.

**Trial registration:**

This study is registered in the Dutch Trial Register (NTR3941). Registered 19 April 2013.

## Background

Worldwide, 65 million people have moderate to very severe chronic obstructive pulmonary disease (COPD). [1] COPD is associated with high burden on society, both in terms of wellbeing of patients and their family as well as economic. Exacerbations of COPD and hospitalizations are responsible for the majority of the COPD-related healthcare costs. [2] Not only to reduce health care costs, but also to maintain patient’s well-being, exacerbations of COPD and hospitalizations should be prevented. [2] This can be achieved by self-management programs. [3–5] The patient’s capacity to self-manage the disease, at least partly, depends on the disease-related knowledge. [6] Next to acquiring and applying skills, self-management programs should focus on increasing the patient’s knowledge to cope with the disease and its related exacerbations. [7] However, disease specific knowledge has proven to be insufficient in about half of the patients with COPD. [8] Besides this, general health knowledge regarding physical activity and nutrition behaviour is also limited in the general older population. [9]

Proxies living together with patients with COPD could support patients in managing their disease. [10] Therefore, they also need health-related and COPD-specific knowledge. Moreover, proxies have an impaired health status themselves, as they are often current smokers and often have (undiagnosed) morbidities. [11] So, they can benefit from health-related knowledge as well. The level of knowledge could be increased by providing education to proxies. Indeed, in caregivers of patients with (severe) mental illness, education proved to increase knowledge, reduce anxiety, [12] and reduce subjective burden. [13] As a consequence, COPD-related knowledge may result in improved caring behaviour. [14] However, the current level of knowledge in resident proxies of patients with COPD remains unknown.

Therefore, the current study aimed to compare health-related knowledge (including COPD-related knowledge) between patients with COPD and their resident proxies. A priori, we hypothesized that both patients and proxies have an incomplete knowledge about COPD.

## Methods

### Study design

The current cross-sectional analysis is part of the Home Sweet Home study, a longitudinal study on the home environment of patients with COPD. [15] This study is approved by the Medical Research Ethics Committees United (MEC-U), the Netherlands (NL42721.060.12/M12–1280), and registered in the Dutch Trial Register (NTR3941). The study protocol and data about health status, morbidities and problematic activities of daily life, were published before. [11, 15, 16]

### Study population

Patients with COPD were recruited by their chest physician or a respiratory nurse specialist during hospital admission or at the outpatient respiratory clinic in four hospitals throughout the southern-eastern part of the Netherlands. In addition, patients who participated in the ‘Chance study’ (NTR3416), [17] met the inclusion criteria of the Home Sweet Home study and were willing to participate in future research were also asked to participate in the current study.

Patients were eligible if they had moderate to very severe COPD (Global initiative for chronic Obstructive Lung Disease (GOLD) grade II to IV); [2] no exacerbation of COPD or hospitalisation within 4 weeks preceding enrolment; and if they had a resident proxy (defined as: a person living together with a patient with COPD, regardless of whether they provide informal care to the patient with COPD). Patients and/or proxies were excluded if they were unable to complete the study questionnaires because of cognitive impairment (Short Blessed Test score ≥ 10 points) [18]; or if they were unable to understand Dutch. Participants were included during the first home visit at least four weeks after a hospital admission or exacerbation, which took place between July 2013 and December 2014. All participants gave written informed consent.

### Measurements

All outcomes were assessed during home visits, including: demographics, level of education (intermediate vocational education or lower and secondary general education or higher), post-bronchodilator spirometry [2], smoking status, reported comorbidities (Charlson comorbidity index) [19], cognitive functioning (Short Blessed Test) [18], presence of (informal) care, and whether patients had followed a pulmonary rehabilitation program.

Knowledge of patients and proxies was assessed using 34 statements about generic health and COPD-related topics. This questionnaire with 34 statements was not validated. However, these 34 statements were all formulated by a multidisciplinary pulmonary rehabilitation team and checked by (inter-)national experts in the field of COPD, in order to establish face validity. All statements were pre-tested in patients with COPD who participated in a pulmonary rehabilitation program, to make sure participants would be able to understand and respond to the statements correctly. All statements could be answered by ‘true’, ‘false’, or ‘do not know’.

### Statistics

Categorical variables are described as frequencies, while continuous variables were tested for normality and are presented as mean and standard deviation (SD). A normal distribution was defined as a skewness or kurtosis ranged between −1.5 and 1.5. [20] To compare continuous variables between patients with COPD and their resident proxies, independent samples t-tests or Mann-Whitney U tests were used, as appropriate. Individual knowledge statements were analysed as an ordinal variable. Ordinal and categorical variables were compared between patients with COPD and their resident proxies using Chi-square tests. The total number of correct, incorrect and ‘do not know’ answers on the knowledge questionnaire were analysed as a continuous variable. The number of correct, incorrect and ‘do not know’ answers on the knowledge questionnaire, stratified for patients with COPD GOLD grade II, III or IV were compared using one-way analysis of variances (ANOVA) with LSD as post hoc test or Kruskal-Wallis test followed by Mann-Whitney U-tests, as appropriate. The percentages of patients who attended pulmonary rehabilitation, stratified for COPD GOLD grade II, III or IV severity levels were compared using chi-square tests. Because of multiple comparisons, the level of significance was set at *p* ≤ 0.01. A multiple regression analysis model was developed to study predictors of patients’ knowledge. The number of correct statements in patients was used as dependent variable and the highest completed education, participation in pulmonary rehabilitation, cognitive functioning (Short Blessed Test), age, and the years since COPD diagnosis were entered as independent variables. A sample size calculation was performed for the primary objective of the Home Sweet Home study, and described in the research protocol. [15] The sample size was estimated using G power. A total of 171 patients and 171 proxies were needed to detect an effect size of 0.25 with a significance of 5% and power of 90%. Although no additional sample size calculation was performed for the objective of the current study, we consider 194 patients and their 194 proxies sufficient. All statistics were performed using SPSS version 20.0.

## Results

### General characteristics

**Fig. 1:**
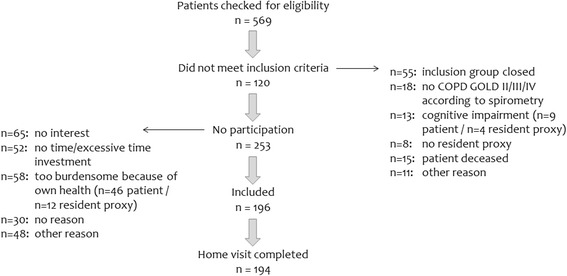
Flow-chart. Abbreviations: COPD = Chronic Obstructive Pulmonary Disease; GOLD = Global initiative for chronic Obstructive Lung Disease

In total, 194 of the 449 eligible patients and their 194 resident proxies were willing to participate and completed the home visit (response rate 43%). Age (*p* = 0.03), gender distribution (*p* = 0.15) and GOLD grade (*p* = 0.20) were comparable between included patients and eligible patients who refused to participate because of various reasons (Fig. 1). [11]

Almost half of the patients (48%) had COPD GOLD grade II, 32% GOLD grade III and 20% GOLD grade IV. Patients’ self-reported time since diagnosed with COPD was 8.7 (7.1) years. Mean age, gender distribution, cognitive functioning and level of education were comparable between patients and their resident proxies (Table 1). Most proxies were married with or partner of the patient, as there were only 3 parent/child relationships between patients and proxies. Almost one third of the resident proxies had a Tiffeneau index below the cut-off value of 70%, which is suggestive for an obstructive airflow limitation. Patients scored significantly more points on the Charlson comorbidity index. Proxies were significantly more often current smokers. Moreover, almost half of the patients participated at least once in pulmonary rehabilitation. In this subgroup, the median time since their last pulmonary rehabilitation program was 24 (3–36) months.

**Table 1:** General characteristics

	Patients with COPD (*n* = 194)	Resident proxies (*n* = 194)	*p*-value
Male, n (%)	102 (52.6%)	87 (44.8%)	0.128
Age (years), mean (SD)^a^	66.0 (8.7)	64.8 (9.7)	0.329
Relationship, n (%)			0.801
- Married or partners	191 (98.5%)	191 (98.5%)	
- Parent/child	3 (1.5%)	3 (1.5%)	
Years living together, mean (SD)	37 (14)	37 (14)	0.877
Tiffeneau Index <70%, n (%)	194 (100%)	56 (29.5%)^†^	<0.001
FEV_1_ (% predicted), mean (SD)^a^	47.2 (17.8)	104.1 (25.6)^†^	<0.001
Charlson comorbidity index, mean (SD)^a^	2.3 (1.4)	1.2 (1.6)	<0.001
Current smoker, n (%)	33 (17.0%)	63 (32.5%)	<0.001
Short Blessed Test (points), mean (SD)	1.6 (2.1)	1.3 (3.0)	0.150
Level of education, n (%)			0.671
- Intermediate vocational education or lower	166 (85.6%)	163 (84.0%)	
- Secondary general education or higher	28 (14.4%)	31 (16.0%)	
Working situation, n (%)			<0.001
- Paid job	17 (8.8%)	40 (20.6%)	
- Retired	97 (50.0%)	78 (40.2%)	
- Household work	23 (11.9%)	45 (23.2%)	
- Unable to work	46 (23.7%)	18 (9.3%)	
- Other (volunteer, or unemployed)	11 (5.7%)	13 (6.7%)	
Receiving care in past 6 months			<0.001
- Informal care, n (%)	32 (16.5%)	3 (1.5%)	
- Care from professional, n (%)	40 (20.6%)	5 (2.6%)	
Participated in a rehabilitation program, n (%)			<0.001
- Pulmonary rehabilitation	84 (43.3%)	7 (3.6%)	
- Other rehabilitation program	24 (12.4%)	23 (11.9%)	

Values expressed as mean (SD) or number of participants (%)

*Abbreviations*: *COPD* Chronic Obstructive Pulmonary Disease, *GOLD* Global initiative for chronic Obstructive Lung Disease

^a^non-parametric statistic tests were used because of skewed data

^†^ *n* = 190

### COPD and health related knowledge

Patients answered on average 22 statements (64.7%) correct and resident proxies 21 statements (61.8%). No significant differences were found in knowledge between patients and their proxies (Table 2). In addition, no differences were found in correct answers (20.6 vs 21.0), incorrect answers (6.7 vs 6.1) and ‘do not know’ responses (6.2 vs 6.8) between proxies with and without a Tiffeneau Index <70% (all *p* > 0.05). Significant differences did exist in the number of correct answers and ‘do not know’ responses between patients who attended pulmonary rehabilitation in the past and those patients who did not (Table 3). Additionally, patients with COPD GOLD grade IV answered significantly more statements correct and significantly less statements with ‘do not know’ compared to patients with COPD GOLD grade II (Table 4). Moreover, less patients with COPD GOLD grade II attended pulmonary rehabilitation previously compared to patients with COPD GOLD grade III and GOLD grade IV. A multiple regression model in patients with COPD, with the number of correct statements as dependent variable, and participation in pulmonary rehabilitation, cognitive functioning (Short Blessed Test), age, the highest completed education, and the years since COPD diagnosis as independent variables was able to explain 33.0% of the variance in correct statements (Table 5). More correct answers were reported by: patients who previously participated in pulmonary rehabilitation, patients with better cognitive functioning, younger patients, patients with a higher level of education, and patients who were diagnosed with COPD longer ago.

**Table 2:** Knowledge of patients with COPD and their resident proxies

	Patients with COPD (*n* = 194)	Resident proxies (*n* = 194)	*p*-value
Knowledge statements			
- Correct answers, mean (SD)	21.6 (4.9)	20.9 (5.4)^†^	0.208
- Incorrect answers, mean (SD)	5.9 (2.5)	6.3 (2.5)^†^	0.130
- ‘Do not know’, mean (SD)^a^	6.5 (4.9)	6.6 (5.4)^†^	0.899

Values expressed as mean (SD)

*Abbreviations*: *COPD* Chronic Obstructive Pulmonary Disease

^a^Non-parametric statistic tests were used because of skewed data

^†^ *n* = 193

**Table 3:** Knowledge of patients who did and did not previously attend pulmonary rehabilitation

	Patients who attended PR (*n* = 84)	Patients who did not attend PR (*n* = 110)	*p*-value
Knowledge statements			
- Correct answers, mean (SD)	23.5 (4.3)	20.0 (4.8)	<0.001
- Incorrect answers, mean (SD)	5.7 (2.6)	6.1 (2.4)	0.337
- ‘Do not know’, mean (SD)	4.7 (4.2)	7.9 (5.0)	<0.001

Values expressed as mean (SD)

*Abbreviation: PR* pulmonary rehabilitation

**Table 4:** Mean values of correct, incorrect and ‘do not know’ answers and percentages of pulmonary rehabilitation attendance in patients with COPD GOLD grade II, GOLD grade III and GOLD grade IV

	Patients with COPD GOLD grade II (*n* = 93)	Patients with COPD GOLD grade III (*n* = 62)	Patients with COPD GOLD grade IV (*n* = 39)	*p*-value
Knowledge statements				
• Correct answers, mean (SD)^a^	19.4 (5.5)^†^	20.4 (5.0)	25.2 (3.3)	0.006
• Incorrect answers, mean (SD)	6.2 (2.5)	6.8 (2.6)	5.8 (2.0)	0.767
• ‘Do not know’, mean (SD)^a^	8.4 (6.1)^†^	6.3 (4.3)	3.1 (2.8)	0.006
Pulmonary rehabilitation				
• Patients who attended PR, n (%)	24 (25.8%)^†,‡^	33 (53.2%)	27 (69.2%)	<0.001

Values expressed as mean (SD)

*Abbreviations*: *COPD* Chronic Obstructive Pulmonary Disease, *PR* pulmonary rehabilitation

^a^Non-parametric statistic tests were used because of skewed data

^†^ *p* < 0.01 vs patients with COPD GOLD grade IV

^‡^ *p* < 0.01 vs patients with COPD GOLD grade III

**Table 5:** Multiple regression model, the predictors of knowledge in patients with COPD

Model	Predictors	Unstandardized Beta	*p*-value
Number of correct statements	Attended pulmonary rehabilitation	3.101	<0.001
	Cognitive functioning (SBT), points	−0.536	<0.001
R^2^ = 0.330	Age, years	−0.123	0.001
	Level of education	0.846	<0.001
	Years since diagnosis COPD	0.135	0.002

*Abbreviations*: *SBT* Short Blessed Test

*n* = 194

Regarding individual statements, two were answered significantly different between patients and proxies, namely: “*The spacers of puffers should be wiped dry after rinsing”* and *“Regular exercises and the intake of milk products will reduce the risk for osteoporosis”* (Table 6). The first statement regarding medication was answered more often correct by patients, while the second statement about general health was answered more often correct by proxies. Nine statements were answered correctly by less than 50% of the patients and proxies.

**Table 6:** Individual knowledge statements of patients and resident proxies

	Correct answers		Incorrect answers		‘Do not know’		
	Patients with COPD	Resident proxies	Patients with COPD	Resident proxies	Patients with COPD	Resident proxies	*p*-value
*COPD in general*							
*1. COPD means Chronic Obstructive Pulmonary Disease. (T)*	86.1%	82.4%	2.6%	2.6%	11.3%	15.0%	0.561
*2. The amount of air that can be blown out quickly (the FEV*_*1*_ *) is reduced in people with COPD. (T)*	59.3%	51.8%	3.1%	2.6%	37.6%	45.6%	0.282
*3. Smoking is the most important cause of COPD. (T)*	80.4%	77.7%	11.3%	10.9%	8.2%	11.4%	0.581
*4. Everyone who has COPD is eligible for lung transplantation. (F)*	53.1%	59.6%	4.6%	3.6%	42.3%	36.8%	0.428
*Living with COPD*							
*5. Anxiety and sadness have a negative influence on the quality of life of people with COPD. (T)*	63.4%	68.4%	16.0%	10.9%	20.6%	20.7%	0.327
*6. Support and understanding for my lung disease from people in my home environment (for example: partner, children and friends) is important. (T)*	94.8%	96.9%	1.0%	0.5%	4.1%	2.6%	0.592
*7. A lung patient who attends the psychologist during pulmonary rehabilitation is insane. (F)*	67.5%	68.9%	4.6%	5.7%	27.8%	25.4%	0.796
*Functioning with COPD*							
*8. My lung function determines which activities I will be able to do at home. (F)*	17.0%	7.8%	71.1%	82.4%	11.9%	9.8%	0.013
*9. A rollator could help a person with COPD to walk further. (T)*	69.6%	59.6%	9.8%	19.7%	20.6%	20.7%	0.019
*10. During strenuous exercise (like climbing 2 stairs) it is better to rest once for a long time than several times for short periods. (F)*	36.1%	29.0%	38.7%	47.2%	25.3%	23.8%	0.203
*11. Leaning forward and bracing with my arms can help to reduce breathlessness. (T)*	32.5%	28.0%	38.7%	36.3%	28.9%	35.8%	0.331
*12. When I lift a heavy shopping bag I should try to breathe out. (T)*	54.6%	54.4%	20.6%	23.8%	24.7%	21.8%	0.663
*COPD and medication*							
*13. The spacers of puffers should be wiped dry after rinsing. (F)*	62.4%*	49.2%	20.6%	21.8%	17.0%*	29.0%	0.010
*14. Shaky hands are a possible side effect of bronchodilators. (T)*	38.1%	35.8%	15.5%	6.7%	46.4%	57.5%	0.011
*15. I will reduce the risk of getting a chest infection by using my lung medication (“puffers”) correctly. (T)*	71.6%	68.9%	12.4%	14.5%	16.0%	16.6%	0.797
*Lung function and oxygen*							
*16. It is safe to use oxygen therapy while cooking with gas. (F)*	58.2%	61.7%	4.1%	3.6%	37.6%	34.7%	0.788
*17. My lung function will change by using long-term oxygen therapy. (F)*	31.4%	26.4%	16.5%	24.9%	52.1%	48.7%	0.114
*18. Breathlessness is always accompanied by low oxygen levels. (F)*	24.7%	22.3%	62.9%	62.7%	12.4%	15.0%	0.688
*Exercising*							
*19. Exercising will improve my lung function. (F)*	9.8%	7.3%	83.0%	87.0%	7.2%	5.7%	0.532
*20. After pulmonary rehabilitation I have to stay active in order to maintain my exercise tolerance. (T)*	91.8%	94.3%	0.5%	0%	7.7%	5.7%	0.437
*21. Daily exercising with my arms and shoulders will make dressing and undressing harder. (F)*	76.3%	66.3%	11.9%	15.0%	11.9%	18.7%	0.082
*22. It is better to avoid exercise because it will strain my lungs too much. (F)*	88.7%	77.7%	6.7%	12.4%	4.6%	9.8%	0.015
*Phlegm*							
*23. I should contact my doctor or nurse when the colour of my phlegm changes (from white to yellow or green) and I experience more symptoms. (T)*	88.7%	89.1%	1.0%	0.5%	10.3%	10.4%	0.848
*24. Phlegm is harmful when swallowed. (F)*	43.8%	48.2%	21.1%	22.8%	35.1%	29.0%	0.444
*General health*							
*25. A “chronic disease” means a disease which heals well. (F)*	83.5%	86.5%	9.3%	6.2%	7.2%	7.3%	0.529
*26. Daily intake of 2 pieces of fruit is recommended. However, 1 piece of fruit may be replaced by 1 glass of orange juice. (T)*	52.6%	59.9%^†^	21.1%	20.8%^†^	26.3%	19.3%^†^	0.222
*27. I can improve my exercise tolerance by strength training. (T)*	89.7%	88.1%	3.1%	1.6%	7.2%	10.4%	0.349
*28. Fresh vegetables are better for my health than frozen or canned vegetables. (F)*	38.7%	35.8%	51.5%	57.0%	9.8%	7.3%	0.477
*29. Regular exercises and the intake of milk products will reduce the risk for osteoporosis. (T)*	79.4%*	90.7%	5.7%	1.6%	14.9%	7.8%	0.006
*30. Stopping smoking reduces the risk of heart disease. (T)*	93.3%	91.2%	1.5%	3.6%	5.2%	5.2%	0.434
*31. Fat is the most important nutrient to build up muscles. (F)*	51.5%	58.0%	15.5%	14.5%	33.0%	27.5%	0.411
*32. When being overweight you have an increased risk for lifestyle diseases, like diabetes and cardiovascular disease. (T)*	91.8%	93.3%	1.0%	3.1%	7.2%	3.6%	0.114
*33. Osteoporosis increases the risk of breaking my hip. (T)*	90.7%	92.7%	1.5%	0.5%	7.7%	6.7%	0.558
*34. ‘Self-management’ means that I do not have to visit a doctor. (F)*	83.5%	72.0%	3.1%	5.7%	13.4%	22.3%	0.025

Values expressed as number of participants (%)

*Abbreviations*: *COPD* Chronic Obstructive Pulmonary Disease, *F* statement is false, *T* statement is true

^†^1 missing: *n* = 194 patients, *n* = 193 proxies

*p < 0.01 vs resident proxies

## Discussion

### Key findings

Patients answered about two third of the 34 statements correct (64%). For proxies this was similar (62%). So, patients and proxies answered on average 17% and 19% of the statements incorrect, and patients and proxies answered on average 19% with ‘do not know’.

### Knowledge in patients with COPD

This study showed that patients answered on average 17% of the statements about COPD and general health incorrect and 19% with ‘do not know’. Statements answered often incorrect were mostly about lung function and functioning with COPD, such as “*Exercising will improve my lung function”* and *“My lung function determines which activities I will be able to do at home”.* Previous studies showed a need for information about COPD and its consequences as well, for both patients and their proxies. [21, 22] The conclusions of Seamark [21] and Wilson [22], together with the results of this study, is sufficient to conclude that education for patients with COPD is currently inadequate and therefore necessary. Patients who were younger, attended pulmonary rehabilitation, had a higher level of education, better cognitive functioning, and with a diagnosis of COPD longer ago had a higher knowledge-level compared to other patients. This is in line with another study, which found that patients who attended a pulmonary rehabilitation program had better understanding about, for instance, the advantages of exercising, in contrast to patients who did not attend a rehabilitation program. [22] On the other hand, patients who attended pulmonary rehabilitation previously, answered only 3 more statements correct than patients who did not attend pulmonary rehabilitation. Considering the total number of 34 statements presented to the patients, this is only a 9% benefit. Moreover, the number of wrong answers remained unchanged. It should be noted that patients with COPD GOLD grade IV answered more statements correct but also attended more often a pulmonary rehabilitation program compared to patients with COPD GOLD grade II, which might explain the results. Furthermore, patients with a very low level of cognitive functioning (Short Blessed Test score ≥ 10 points) were excluded from the present study. Yet, it is also well-known that patients with COPD are most often older and have lower levels of education compared to the general population. [23] Moreover, poor COPD outcomes (like hospitalization and mortality) are more likely to be found in patients of low socioeconomic status, compared to patients of high socioeconomic status. [24] Additionally, about 85% of the patients and proxies had a low level of education, and the current analysis showed that these patients reported less correct answers on the statements. Therefore, the knowledge gap in these proxies and patients with COPD could be the result of the low educational levels or low socioeconomic status. However, a non-COPD control group should be included to investigate this in depth. Indeed, Friis and colleagues, [23] found that people with long-term conditions reported more difficulties with understanding health information compared with the general population.

Another remaining question is if current education programs are sufficient in this group of patients. However, we did not study the quality of the provided education within rehabilitation programs. A systematic review showed the wide variation in the content and method of delivery of educational interventions in patients with COPD. [25] In the “official American Thoracic Society/European Respiratory Society statement: key concepts and advances in pulmonary rehabilitation”, a list of relevant educational topics is provided. [3]

### Knowledge in resident proxies of patients with COPD

Not only patients, but also proxies answered less than two thirds of the statements correctly. In previous literature proxies identified five areas of learning, namely: 1) understanding breathlessness; 2) managing anxiety and panic; 3) helpful and safe levels of activity; 4) maintaining quality of life; and 5) knowing what to expect in the future. [26] Indeed, a perfect example of a statement which is often answered incorrect is: ‘*It is better to avoid exercise because it will strain my lungs too much*’. Believing this statement could lead to overprotective behaviour when proxies let patients avoid exercising and take over activities. Except for the fact that this could lead to frustrations between a couple, [27] and distress in patients, [28] it could also lead to a less active way of living for patients. [27] Therefore, education should be provided to patients together with their proxies. Indeed, studies showed that education sessions for patients and proxies together were beneficial in terms of improved coping strategies, [29] strengthening the relationship and a decreased burden. [30] Moreover, education is needed to arrange lifestyle changes. [31]

### Future perspectives

The current study shows an incomplete health related knowledge in patients with COPD and their resident proxies, thus leaving room for further improvements. Small differences were found in the total number of correct answered statements, between patients who attended a pulmonary rehabilitation program and patients who did not, and between patients with GOLD grade II compared to GOLD grade IV. Thus, education should be made available in all subgroups of patients with COPD in primary, secondary and tertiary care. Also resident proxies should be able to attend education sessions about general health and COPD, because they have an incomplete knowledge as well. As mentioned before, the assessed statements were not validated. Therefore, further research is necessary to validate these statements. On the other hand, the present study showed the specific educational needs of patients and proxies, which together with the list of relevant educational topics from the official ATS/ERS statement, [3] could be used as a basis to provide education sessions.

Cognitive functioning proved to be a predictor of patients’ knowledge about general health and COPD. Although half of the written material for educational interventions is adapted to the patients’ level of literacy, [25] it remains unknown whether other education sessions are adjusted to the patients’ cognitive functioning. Moreover, a study in chronic pain patients suggested that an assessment of the patient’s learning style might lead to a better fit of the patient education. [32] Additionally, a study in the primary care setting showed that increasing knowledge alone provided no additional health benefit compared to usual care. [33] Therefore, more knowledge should be gained about the use of different learning styles, teaching methods and dyadic approaches in patients with COPD and their proxies, especially regarding the effects on their knowledge level and their capacity to self-manage their disease.

### Methodological considerations

The present study has some limitations. First, the response rate was 43%. Unknown differences between participants and eligible patients refusing participation may be present. For instance, patients with little knowledge could have refused participation, so the current study overestimated the knowledge of participants. On the other hand, perhaps patients with a higher level of knowledge could have refused participation because they did not see any additional benefit in participation. Second, we did not perform a sample size calculation for the objective of this study because no preliminary data were available concerning differences in general health and COPD specific knowledge between patients with COPD and their resident proxies. Future studies should validate the knowledge statements and define a minimum clinical important difference. Third, we did not include a control group of couples from the general population who were matched for age and education. Therefore, comparisons with the non-COPD population, regarding health related knowledge, could not be made. In addition, we did not include a group of health care professionals to check their knowledge about COPD and general health. Moreover, we also did not include a group of patients with COPD without a resident proxy (so, patients who lived alone). Therefore, comparisons with this COPD populations could also not be made. Fourth, participants’ knowledge was not assessed using a validated questionnaire, so further research is necessary to validate the questionnaire. However, existing questionnaires focus more on specific disease-related knowledge, [34–36] while the current statements were also general in nature. In addition, these statements were formulated by a multidisciplinary pulmonary rehabilitation team, checked by (inter-)national experts and pre-tested in patients with COPD.

## Conclusions

The present study showed that patients and proxies answered about two third of the 34 statements correct, so both patients and their proxies have an incomplete knowledge which leaves room for further improvements. Therefore, education about general health and COPD should be provided to clinically stable outpatients with COPD and their proxies, regardless of the patients’ disease severity.

## Abbreviations

COPD: Chronic obstructive pulmonary disease
GOLD: Global initiative for chronic Obstructive Lung Disease
MEC-U: Medical Research Ethics Committees United
SD: Standard deviation
SPSS: Statistical Package for the Social Sciences
